# Cloning and Expression of Cockroach α7 Nicotinic Acetylcholine Receptor Subunit

**DOI:** 10.3389/fphys.2020.00418

**Published:** 2020-05-07

**Authors:** Alison Cartereau, Emiliane Taillebois, Balaji Selvam, Carine Martin, Jérôme Graton, Jean-Yves Le Questel, Steeve H. Thany

**Affiliations:** ^1^LBLGC, UPRES EA 1207-USC INRA 1328, Université d’Orléans, Orléans, France; ^2^Roger Adams Laboratory, University of Illinois at Urbana-Champaign, Urbana, IL, United States; ^3^CEISAM-UMR CNRS 6230, Faculté des Sciences et des Techniques, Université de Nantes, Nantes, France

**Keywords:** nicotinic receptor, insect, α7 subunit, acetylcholine, nicotine, neonicotinoid

## Abstract

Understanding insect nicotinic acetylcholine receptor (nAChR) subtypes is of major interest because they are the main target of several insecticides. In this study, we have cloned a cockroach Pameα7 subunit that encodes a 518 amino acid protein with futures typical of nAChR subunit, and sequence homology to α7 subunit. Pameα7 is differently expressed in the cockroach nervous system, in particular in the antennal lobes, optical lobes and the mushroom bodies where specific expression was found in the non-compact Kenyon cells. In addition, we found that cockroach Pameα7 subunits expressed in *Xenopus laevis* oocytes can assemble to form homomeric receptors. Electrophysiological recordings using the two-electrode voltage clamp method demonstrated that nicotine induced an I_*max*_ current of −92 ± 27 nA at 1 mM. Despite that currents are low with the endogenous ligand, ACh, this study provides information on the first expression of cockroach α7 homomeric receptor.

## Introduction

Insect neuronal nicotinic acetylcholine receptors (nAChRs) are of particular interest because they are the main target of neonicotinoid insecticides, which are important in agriculture and veterinary medicine for controlling insect pests, and preventing transmission of insect borne diseases (Casida, 2009). In general, the pharmacological properties of insect native nAChRs are studied using electrophysiological approaches, with isolated neurons expressing nAChR subtypes (Thany et al., 2007; Barbara et al., 2008; Salgado, 2016). Cockroach neurons from thoracic ganglia and dorsal unpaired median (DUM) neurons are currently used to characterize the pharmacological properties of insect native nAChR subtypes, and the mode of action of neonicotinoid insecticides (Courjaret and Lapied, 2001; Courjaret et al., 2003; Calas-List et al., 2012; Salgado, 1998; Salgado and Saar, 2004; Thany et al., 2008). Using cockroach thoracic ganglia, two α-bungarotoxin (α-Bgt)-sensitive nAChR subtypes were characterized: nAChD and nAChN. nAChD was desensitizing, and selectively inhibited by IMI, and nAChN was non-desensitizing, and selectively inhibited by methyllicaconitine (MLA) (Salgado, 2016; Salgado and Saar, 2004). Moreover, nAChD receptors are potently inhibited by neonicotinoid insecticides whereas nAChN are activated by neonicotinoids (Salgado and Saar, 2004). α-Bgt-sensitive and -insensitive nAChR subtypes were also found in the DUM neurons. Two α-Bgt-insensitive receptors were identified as nAChR1 and nAChR2. nAChR1 was sensitive to imidacloprid, and selectively blocked by d-tubocurarine (d-TC), and nAChR2 was inhibited by mecamylamine (MEC) (Courjaret and Lapied, 2001; Courjaret et al., 2003; Thany et al., 2008; Bodereau-Dubois et al., 2012). Unfortunately, although detailed information is available concerning the pharmacological properties of cockroach native nAChR subtypes, the subunit combination of these receptors is unknown.

Genes encoding insect nAChR subunits were cloned from several insect species, including the fruit fly *Drosophila melanogaster*, the honey bee *Apis mellifera* and the mosquito *Anopheles gambiae* for which the genome is known. From comparison of the insect and vertebrate nAChR subunits it appeared that the monophyletic group including drosophila Dα5, Dα6 and Dα7, is closely related to mammalian α7 subunit (Thany et al., 2007). This group is of specific interest because mammalian α7 subunits form homomeric receptors which are currently used to study the pharmacology and functional properties of nAChR subtypes (Gill et al., 2013; Delbart et al., 2018). Binding investigations using nicotinic agonists showed that α7 nAChR mediates inward currents sensitive to nAChR antagonists such as α-Bgt or MLA when applied coincidentally with agonists, or pre-exposed to antagonists before agonist application (Cuevas et al., 2000; Virginio et al., 2002; Zhao et al., 2003). A previous study suggests that members of this group could form homomeric receptors when they are expressed in heterologous systems (Lansdell et al., 2012). Indeed, despite there having been only limited success in expressing insect nAChR subunits, a direct expression of the drosophila Dα7 subunit in *Xenopus laevis* oocytes formed a functional receptor when it was co-expressed with the chaperone resistant to inhibitors of acetylcholinesterase (RIC-3) (Lansdell et al., 2012). However, no specific α-Bgt binding was detected (Lansdell et al., 2012), suggesting that drosophila α7 receptors could be insensitive to α-Bgt. Moreover, the drosophila Dα5 subunit was able to form a homomeric α-Bgt-sensitive receptor when co-expressed with RIC-3 (Lansdell and Millar, 2004; Lansdell et al., 2012). Thus, the pharmacological properties of the α7 monophyletic group seemed to be more complex.

In the present study, we report the cloning and expression of a cockroach Pameα7 subunit in the *Xenopus laevis* oocytes. We show that Pameα7 subunit can form a functional receptor in the *Xenopus* oocytes.

## Materials and Methods

### Insects

All experiments were performed with cockroach *Periplaneta americana* laboratory-reared insects.

### Compounds

ACh, nicotine, MLA, MEC, d-TC and atropine were purchased from Sigma Chemical Co. (St Quentin, France). α-Bgt was purchased from Biotrend (Köln, Germany).

### Bioinformatic Analysis

Sequence alignment were made with BioEdit software and deduced amino acid sequences were analyzed using the ClustalW program (Thompson et al., 1994). The location of the functional domains was determined using TMHMM 2.0 software (Moller et al., 2001). nAChR subunit sequences used for phylogenetic analysis were downloaded from GenBank database^1^. A phylogenetic tree was constructed using neighbor-joining statistical method (Saitou and Nei, 1987) with Bootstrap test at 1,000 replications and p-distance as substitution model. Branches corresponding to partitions reproduced in less than 50% bootstrap replicates were collapsed. The *D. melanogaster* GABAa subunit was used as outgroup. Analyses were conducted with the MEGA6 program (Tamura et al., 2013).

### RT-PCR Amplification, Cloning and Sequencing of the Cockroach Pameα7 Subunit

Total RNA was isolated from adult brain using the RNeasy Mini Kit (Qiagen). RT-PCR and cDNA cloning was performed as follows: first-strand cDNA was product from 2 μg of total RNA, incubated at 65° for 5 min in the presence of 100 ng oligodT, 0.5 mM dNTP, 10 mM DTT, 1x RT buffer. After adding 1 μl of Superscript II RT (Invitrogen, Carlsbad, CA, United States), the reaction was proceeded at 42° for 50 min and at 70° for 10 min. The complete Pameα7 cDNA sequence was amplified using the following sense and antisense primers: AAGGATCCCCAACCATGGA GTCAACAGCAGCCTCCGA (sense) and CAAAATCTAGATTACGTCACGATGATGTG GGGCG (antisense). PCR amplification conditions were: 94°C 2 min, 30 cycles (94°C for 30 s, 65°C for 30 s, 72°C for 1 min 40 s) and 72°C for 5 min. PCR products were cloned in pGem vector (pGem T-easy vector system, Promega) and sequenced.

### Semi-Quantitative PCR Amplification

For semi-quantitative PCR amplification, the following primers were used GATGGCTTCTCTTCGTCTGC (sense) and CAGCTACCGCTATCCCTGAC (antisense) for Pameα7 subunit. CTGACCCTTAAATACCCCATTG (sense) and CACAATTTCTCGTTCGGCAGTG (antisense) for actin. PCR was performed in a total volume of 25 μl containing 1 μl of RT products, 0.4 μM of each primer, 0.2 mM desoxyribonucleotide triphosphates (dNTP), 1.5 mM MgCl_2_ and 0.125 μl of Taq polymerase (Invitrogen, Carlsbad, CA, United States). The following PCR conditions were used: 20 cycles at 95°C for 30 s, 58°C for 30 s, 72°C for 1 min and a final elongation of 72°C for 5 min. Data were analyzed using image J software. Pameα7 expression level in each sample was normalized with the corresponding actin expression (Taillebois et al., 2014).

### *In situ* Hybridization

*In situ* hybridization on cryostat frontal sections was performed with digoxigenin-labeled RNA probes (Sigma-Aldrich, France) as described previously (Thany et al., 2003; Thany and Gauthier, 2005). A cDNA fragment of 1.6 Kb corresponding to Pameα7 was cloned in pCR 4-TOPO vector (ThermoFisher Scientific, France) with HindIII and XhoI restriction enzymes. After linearization with NotI, *in vitro* transcription was performed with T7 RNA polymerase to generate antisense DIG-labeled RNA probes.

### Preparation of cDNA for Expression in the Xenopus Oocytes

Cockroach Pameα7 (GenBank accession number: JX466891) subunit was cloned into XbaI/BamHI (Invitrogen, Carlsbad, CA, United States) digested pGEM-HEJUEL plasmid (Provided by Prof. Olaf Pongs, Institute for Neural Signal Transduction, Germany, to Prof Christian Legros, University of Angers, France) as previously described (Bourdin et al., 2015). pGEM contains both 5′ and 3′ UTR from the *Xenopus* beta-globin gene, allowing high expression of foreign protein in *Xenopus* oocytes.

### Oocyte Injection in the Xenopus Laevis Oocytes

*Xenopus laevis* oocytes were obtained from the CRB xenope, University of Rennes, France. The CRB xenope is a French national platform dedicated to xenopus breeding for experimental research. *Xenopus laevis* oocytes were stored in a standard oocyte saline solution (SOS) of the following composition: in mM, 100 NaCl, 2 KCl, 1 MgCl2, 1.8 CaCl_2_ and 5 HEPES, pH 7.5. Stage V and VI oocytes were harvested and defolliculated after treatment with 2 mg/ml collagenase IA (Sigma, France) in Ca^2+^-free SOS solution, supplemented with 0.8 mg/ml trypsin inhibitor. Defolliculated oocytes were injected with 2 ng of α7 cDNA cloned in pGEM (Couturier et al., 1990; Ihara et al., 2003). Injected oocytes were maintained at 18°C in SOS solution supplemented with penicillin (100 U/ml), streptomycin (100 mg/ml), gentamycin (50 mg/ml) and sodium pyruvate (2.5 mM).

### Voltage-Clamp Recordings

Currents were recorded 4 days after injection, using two microelectrodes filled with 3 M KCl. The oocyte membrane potential was held at −80 mV (Taylor-Wells et al., 2017), and perfused continuously with recording buffer at room temperature (20–22°C). To suppress potential endogenous muscarinic responses, saline solution containing 0.5 μM atropine was employed (Matsuda et al., 1998, 2000). The dose response curves were estimated by using increasing concentrations of the compounds on the same oocyte. Oocytes were challenged with a test compound at 5 min intervals to minimize receptor desensitization (Ihara et al., 2003). To assess the pharmacological profile of these receptors, experiments were conducted with different antagonists. Experimental data was digitized with a Digidata-1322A A/D converter and then analyzed with pCLAMP (Molecular Devices, Union City, CA, United States). All compound solutions were prepared using the recording buffer.

### Statistical Analysis

For statistical analysis of Pameα7 expression levels, one-way ANOVA and Bonferroni *post hoc* test were employed. All currents were shown as mean ± SEM and analyzed using Prism 7 (GraphPad Software, La Jolla, CA, United States). Note that for all compounds, experiments were also performed on non-injected oocytes to avoid native responses (data not shown). Oocytes were assigned to each group without knowledge of the treatments (blinded). The dose response curves were derived from the fitted curve following the equation: *Y* = I_*min*_ + (I_*max*_−I_*min*_)/(1 + 10^(log(EC50 X)H)^) where *Y* is the normalized response, I_*max*_ and I_*min*_ are the maximum and minimum responses, H is the Hill coefficient, EC_50_ is the concentration giving half the maximum response and *X* is the logarithm of the compound concentration. For the electrophysiological recordings, “*n*” represents the number of experiments. Thus, currents were analyzed using the Kruskal-wallis one-way ANOVA and Bonferroni *post hoc* test. *P* < 0.05 was the minimum level of significance.

## Results

### Cloning and Expression Pattern of Cockroach α7 Subunit in the Nervous System

**FIGURE 1:**
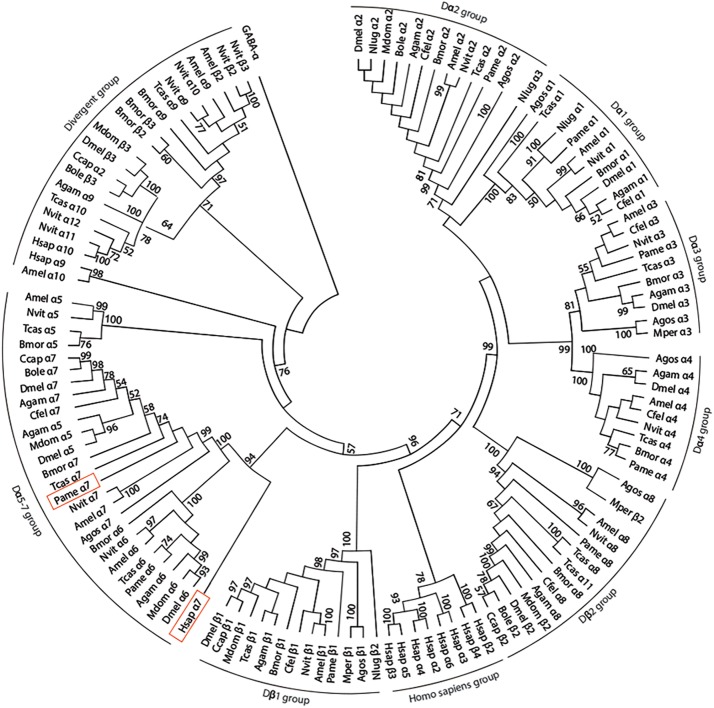
Phylogenetic tree showing relationships of alpha 7 nAChR subunit protein sequence of the cockroach *P. americana* and its orthologs in several insect species and human. The bootstrap support value (%) based on 1,000 replicates are shown when higher than 50%. The *D. melanogaster* GABA_*A*_ subunit (accession number AAA28556.1) was used as outgroup. Accession sequence identifiers are as follows: ***Anopheles gambiae***: Agamα1 (AAU12503.1), Agamα2 (AAU12504.1), Agamα3 (XP_310786.3), Agamα4 (XP_566274.3), Agamα5 (XP_314691.2), Agamα6 (XP_308042.3), Agamα7 (XP_309153.3), Agamα8 (XP_311925.3), Agamα9 (XP_310203.3), Agamβ1 (XP_309158.3); ***Aphis gossypii***: Agosα1 (AAM94383.1), Agosα2 (AAM94382.1), Agosα3 (ABR21379.1), Agosα4 (ABR21380.1), Agosα7 (AFM78640.1), Agosα8 (BBA21164.1), Agosβ1 (AAM94384.1); ***Apis mellifera***: Amelα1 (NP_001091690.1), Amelα2 (NP_001011625.1), Amelα3 (NP_001073029.1), Amelα4 (NP_001091691.1), Amelα5 (AJE70263.1), Amelα6 (NP_001073564.1), Amelα7 (AJE70265.1), Amelα8 (NP_001011575.1), Amelα9 (NP_001091694.1), Amelα10 (XP_392070.3), Amelβ1 (NP_001073028.1), Amelβ2 (NP_001091699.1); ***Bactrocera oleae***: Boleα2 (XP_014100082.1), Boleα7 (XP_014102300.1), Boleβ2 (XP_0111204502.1), Boleβ3 (XP_014090987.1); ***Bombyx mori***: Bmorα1 (ABV45511.1), Bmorα2 (ABV45512.1), Bmorα3 (ABV45513.1), Bmorα4 (ABV45514.1), Bmorα5 (ABV45516.1), Bmorα6 (NP_001091830.1), Bmorα7 (ABV45520.2), Bmorα8 (ABV45521.1), Bmorα9 (ABV45523.1), Bmorβ1 (NP_001166819.1), Bmorβ2 (NP_001103400.1), Bmorβ3 (NP_00110341.1); ***Ceratitis capitata***: Ccapα2 (XP_004536261.1), Ccapα7 (JAB87466.1), Ccapβ1 (XP_012156453.1), Ccapβ2 (XP_012162675.1); ***Drosophila melanogaster***: Dmelα1 (CAA30172.1), Dmelα2 (NP_524482.1), Dmelα3 (CAA75688.1), Dmelα4 (CAB77445.1), Dmelα5 (AAM13390.1), Dmelα6 (NP_723494.2), Dmelα7 (CAD86936.1), Dmelβ1 (P04755.1), Dmelβ2 (CAA39211.1), Dmelβ3 (NP_525098.1); ***Musca domestica***: Mdomα2 (ABD37617.1), Mdomα5 (ABY40460.1), Mdomα6 (ABJ09669.1), Mdomβ1 (XP_005180169.1), Mdomβ2 (XP_005185796.1), Mdomβ3 (ABY40465.1); ***Myzus persicae***: Mperα3 (CAB52297.1), Mperβ1 (XP_022165274.1), Mperβ2 (XP_022167599.1); ***Nasonia vitripennis***: Nvitα1 (ACY82683.1), Nvitα2 (ACY82684.1), Nvitα3 (ACY82685.1), Nvitα4 (ACY82686.1), Nvitα5 (ACY82688.1), Nvitα6 (ACY82689.1), Nvitα7 (ACY82692.1), Nvitα8 (ACY82693.1), Nvitα9 (ACY82694.1), Nvitα10 (ACY82695.1), Nvitα11 (ACY82696.1), Nvitα12 (ACY82697.1), Nvitβ1 (ACY82698.1), Nvitβ2 (ACY82699.1), Nvitβ3 (ACY82700.1); ***Nilaparvata lugens***; Nlugα1 (AAQ75737.1), Nlugα2 (AAQ7574101), Nlugα3 (AAQ75739.1), Nlugβ2 (AAQ75742.2); ***Periplaneta americana***: Pameα1 (AKV94620.1), Pameα2 (AKV94621.1), Pameα3 (AKR16132.1), Pameα4 (AFA28129.1), Pameα6 (AKV94622.1), Pameα7 (MK790056), Pameα8 (AFA28130.1), Pameβ1 (AKV94624.1); ***Tribolium castaneum***: Tcasα1 (ABS86902.1), Tcasα2 (ABS86903.1), Tcasα3 (ABS86904.1), Tcasα4 (ABS86905.1), Tcasα5 (ABS86907.1), Tcasα6 (ABS86908.1), Tcasα7 (ABS86911.1), Tcasα8 (ABS86912.1), Tcasα9 (ABS86913.1), Tcasα10 (ABS86914.1), Tcasα11 (ABS86915.1), Tcasβ1 (ABS86916.1); ***Homo sapiens***: Hsapα2 (AAB40109.1), Hsapα3 (AAA59942.1), Hsapα4 (AAA64743.1), Hsapα5 (AAA58357.1), Hsapα6 (AAB40113.1), Hsapα7 (CAA49778.1), Hsapα9 (CAB65091.1), Hsapα10 (CAC20435.1), Hsapβ2 (CAA37320.1), Hsapβ3 (CAA47851.1), Hsapβ4 (CAA48336.1); ***Ctenocephalides felis***: Cfelα1 (ABB42999), Cfelα2 (ABB43000), Cfelα3 (ABB43001), Cfelα4 (ABB43003), Cfelα7 (ABB43004), Cfelα8 (ABB43002), Cfelβ1 (ABB43005).

**FIGURE 2:**
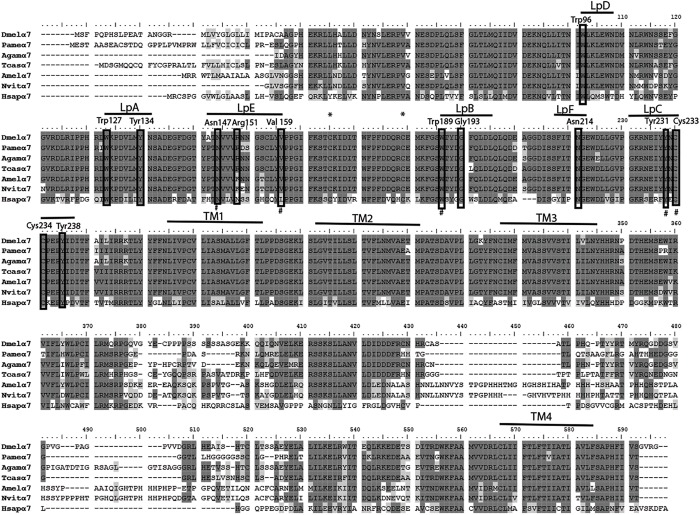
Alignment of nAChR α7 subunit protein sequences of the cockroach *P. americana* with its orthologs in several insect species and human. The loops (LpA-F) involved in ligand binding and transmembrane motifs (TM1-4) forming the ion channel are indicated. Sites of cysteine residues involved in the Cys-loop are marked with asterisk; the vicinal cysteine residues characteristic of alpha-type and the key residues are shown in frame. Alignment was done with drosophila sequence as reference, identical residues (dark gray shading) and similar residues (light gray shading) are indicated.

**FIGURE 3:**
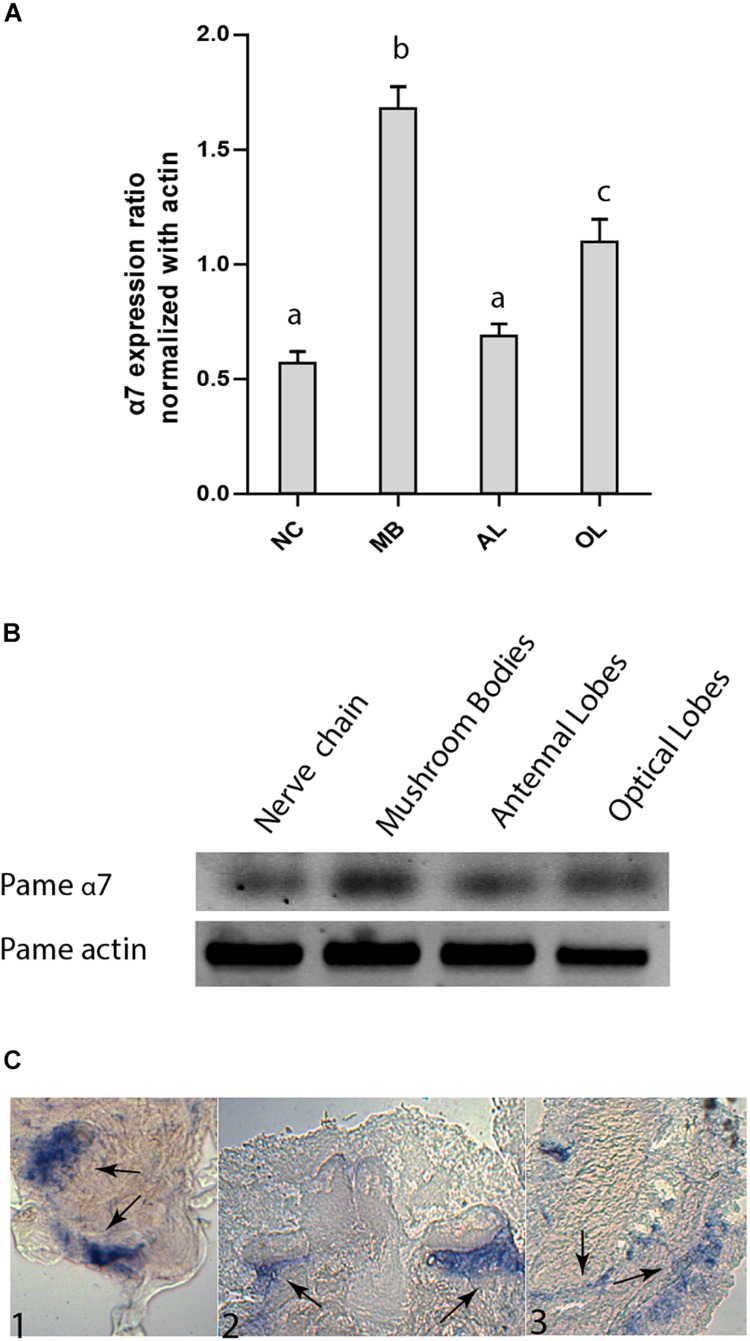
Expression pattern of Pameα7 subunit in the cockroach nervous system. **(A)** Expression level of Pameα7 using semi-quantitative RT-PCR experiments. Data are shown as expression ratio normalized with actin. Expression pattern was determined in the nerve chain (NC), antennal lobes (AL), mushroom bodies (MB) and optical lobes (OL). Significant differences are designated by different letters. **(B)** Electrophoresis showing Pameα7 and actin (used as quantitative and positive control). Dara are the mean of four independent experiments. Significant difference was determined using one-way ANOVA and Bonferroni *post hoc* test. All statistical analyses were added in Supplementary Figure S1C. **(C)** *In situ* hybridization of Pameα7 mRNA on cockroach adult brain. Expression is detected in the antennal lobes (C1), the non-compact Kenyon cells of the mushroom bodies (C2) and the optic lobes (C2).

We have amplified by a nested PCR approach using putative cockroach *Periplaneta americana* α7 subunit sequences (JX466891 and JF731242) available in the GenBank database a full Pameα7 cDNA sequence. Two independent clones were obtained and sequenced, one encoded for a truncated form of Pameα7 (data not shown) and a complete cDNA sequence of Pameα7 subunit (GenBank accession number MK790056) with 1554 bp in length (Cartereau, 2018). This ORF encoded a protein of 518 amino acids with a predicted molecular weight of 58.02 kDa, and an estimated p*I* of 5.45. Comparison of our cloned sequence with putative cockroach α7 sequences available in the GenBank database revealed 98% sequence homology. Additional putative subunit sequences of the cockroach were identified and help us to propose a phylogenetic tree and homogenate nomenclature (Figure 1). Phylogenetic analysis with insect and mammalian nAChR subunits demonstrated that Pameα7 was included in the Dα5-Dα7 group which was defined as closed to the mammalian α7 subunit. The present Pameα7 subunit nomenclature takes this fact into account. We also found two distinct clusters formed by insect β subunits and a divergent cluster formed by nAChR subunits from different insect species (Figure 1). Moreover, amino acid sequence alignment showed that Pameα7 has features typical of the α subunit nAChR family. It contains the functional domains and key amino acids for agonist binding. The four hydrophobic putative transmembrane domains TM1-TM4, the two adjacent cysteines, and extracellular loops (LpA-F) as well as key amino acid residues (Asn147, Trp189, Tyr231,Cys233-234) (Shimomura et al., 2004; Yao et al., 2009; Wu et al., 2015) which are highly conserved between insect and human α nAChR subunits. Except for Val159 which seemed specific to insect species. In addition, the amino acid sequences between TM3 and TM4 appears highly variable (Figure 2). To further investigate the expression of Pameα7 in the cockroach nervous system, we compared its expression level in several nervous tissues (Figures 3A,B). Semi-quantitative PCR experiments highlighted a strong expression in mushroom bodies and optical lobes compared to antennal lobes and nerve chain. In the MBs, using Pameα7-specific RNA probes, we found an expression in outer Kenyon cells of the MBs, in the cells between the lamina and the lobula, and in some cells of the antennal lobes (Figure 3C). Substantial analyses comparing RT-PCR conditions after several PCR cycles were added in the Supplementary Figures S1A,B and Supplementary Table S1).

### Expression of Pameα7 Subunit in *Xenopus laevis* Oocytes

**FIGURE 4:**
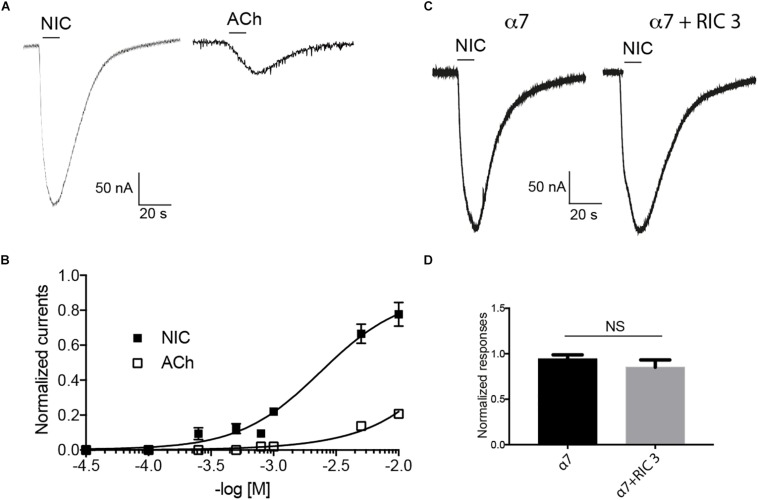
Effect of nicotine (Nic) and acetylcholine (ACh) on cockroach α7 nAChRs expressed in *Xenopus laevis* oocytes. **(A)** Typical example of nicotine- and acetylcholine-induced currents. Currents are recorded at 10 mM nicotine or acetylcholine, respectively. **(B)** Dose-response curve are represented for nicotine and acetylcholine. Data are normalized to 10 mM nicotine and each point represents a mean ± S.E.M of *n* = 12 tested oocytes. **(C,D)** Nicotine (10 mM) evoked currents recorded after the expression of cockroach α7 subunit alone or with rat RIC-3. Each histogram represents mean ± S.E.M of *n* = 10 oocytes. NS = no significant difference using one-way ANOVA and Bonferroni *post hoc* test.

**FIGURE 5:**
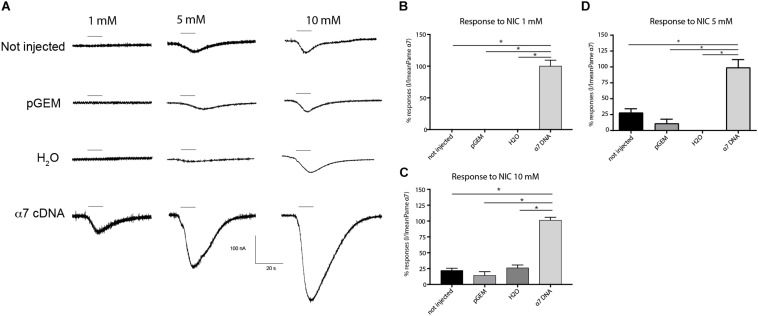
Comparative effect of nicotine on different experimental conditions. **(A)** Typical examples of nicotine-induced currents on not injected oocytes (native oocytes), oocytes with only water, oocytes with pGem vector and oocytes with α7 cDNA. Bars indicate 10 s application of nicotine at 1, 5 or 10 mM. **(B–D)** Histograms summarize applications of nicotine at different experimental conditions. Each histogram represents *n* = 12 oocytes, *p < 0.05 using One-way ANOVA and Bonferroni *post hoc* test.

**FIGURE 6:**
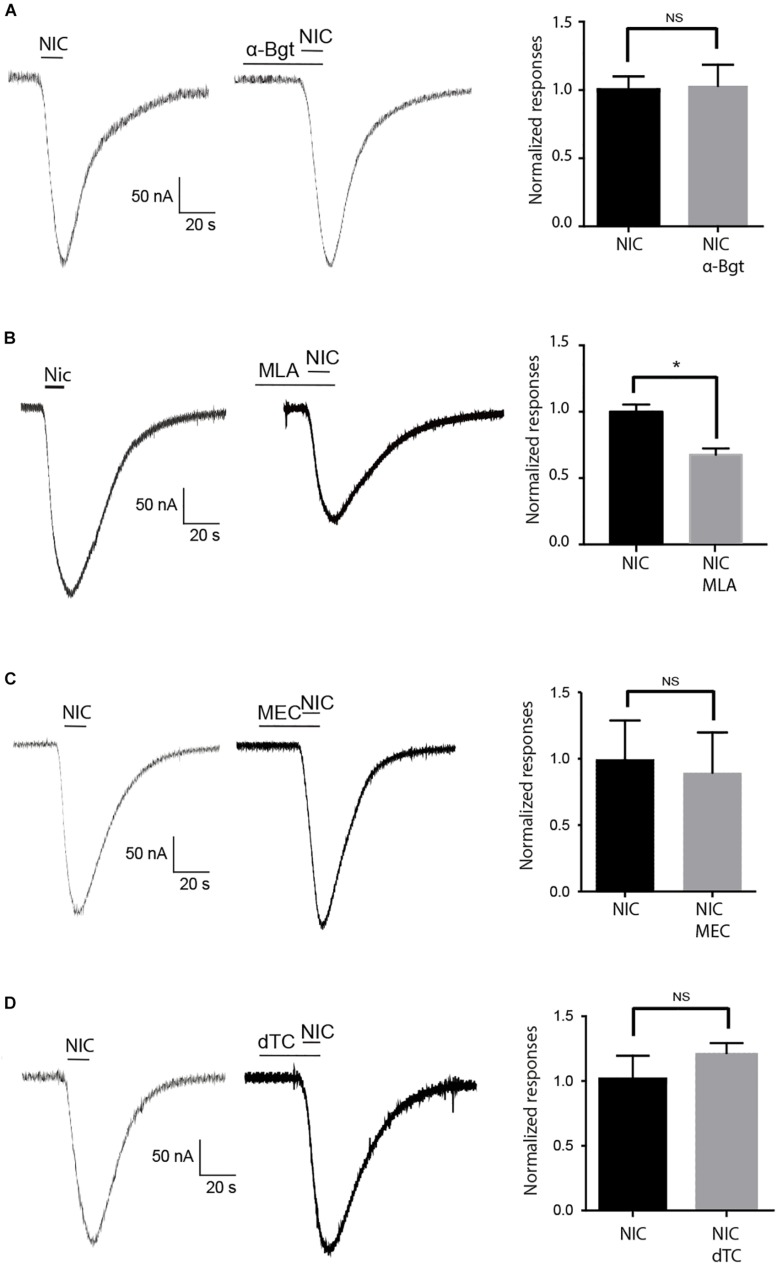
Effect of nAChR antagonists on nicotine evoked currents. **(A)** 5 min pretreatment with 10 μM α-Bgt, **(B)** 10 μM MLA, **(C)** 5 μM mecamylamine (MEC), and **(D)** 10 μM d-TC. Bars indicate application of 10 s nicotine at 10 mM. Histograms illustrate the effect of each antagonist on nicotine-evoked currents. In each case, data are mean ± S.E.M of *n* = 12 oocytes, *p < 0.05 using one-way ANOVA and Bonferroni *post hoc* test. NS = no significant.

The functional expression of the cockroach α7 homomeric receptor was first studied using direct expression of the Pameα7 subunit in *Xenopus laevis* oocytes. The functional expression of cockroach α7 homomeric receptor was first studied using direct expression of α7 subunit in the *Xenopus laevis* oocytes. ACh and nicotine induced inward currents but nicotine activated Pameα7 receptors in a dose-dependent manner and the maximum responses with nicotine were greater than the maximum responses evoked by ACh (Figures 4A,B). At 10 mM, the max currents for nicotine and ACh were −212 ± 0.13 and −56 ± 09 nA, respectively. The EC_50_ for Nicotine was 790 μM whereas for ACh, we were not able to calculate it. Co-expression of cockroach Pameα7 subunit with a rat RIC-3 did not enhance or change the response induced by nicotine (Figures 4C,D, *n* = 18, *p >* 0.05). We proposed that rat RIC-3 is not necessary for the functional expression of Pameα7 receptor and that Pameα7 subunit can form a functional homomeric receptor alone. Moreover, we found also that Pameα7 cDNA formed a functional receptor compared to Pameα7 cRNA (see Supplementary Figure S1C). Thus, we used Pameα7 cDNA, as described in previous studies (Hurst et al., 2005; Moroni et al., 2006; Alcaino et al., 2017). In addition, we decided to select as a test concentration 10 mM nicotine, in consistency with the conditions used for cockroach DUM neurons expressing nAChR subtypes (Courjaret and Lapied, 2001; Courjaret et al., 2003) and because currents induced by ACh were low to conduct a robust analysis. We then tested the effect of nicotine on oocytes containing water and pGem vector. As illustrated in Figure 5, despite that native currents were recorded when we used 5 or 10 mM nicotine, currents induced following 10 s application of 1 mM nicotine demonstrated that Pameα7 cDNA injection expressed a functional receptor in the oocytes. The percentage of successful expression of cockroach Pameα7 receptors that respond to nicotine applications was around 75% (*n* = 120 tested oocytes at 1 mM nicotine). The I_*max*_ values at 1 mM nicotine was −92 ± 27 nA. Moreover, bath application of 10 μM α-Bgt, did not block or reduce nicotine-evoked currents (Figure 6A, *n* = 10 cells, *p* > 0.05, one-way ANOVA and Bonferroni *post hoc* test) but MLA, a potent specific nicotinic antagonist of vertebrate neuronal α7 nAChRs (Davies et al., 1999), reduced 24% of the nicotine evoked currents (Figure 6B, *n* = 12, *p* < 0.05, one-way ANOVA and Bonferroni *post hoc* test). Additional data were performed to investigate if MLA reduced currents on not injected eggs. MLA has no effect on not injected oocytes (see Supplementary Figure S1D). In addition, no blocking or reduction of nicotine currents was found with 5 μM MEC (Figure 6C, *n* = 8, *p* > 0.05, one-way ANOVA and Bonferroni *post hoc* test) or 10 μM d-TC (Figure 6D, *n* = 8, *p* > 0.05, one-way ANOVA and Bonferroni *post hoc* test).

## Discussion

We have cloned using putative sequence available in the GenBank database a DNA fragment corresponding to the cockroach Pameα7. The cDNA for Pameα7 encodes a protein sequence of 518 amino acids which is closely related to the human α7 subunit. Temporal and spatial expression of Pameα7 mRNA demonstrated a specific expression in the MBs. This tissue specific expression was also found with other insects using *in situ* hybridization of transcripts from the honey bee Amelα7. Indeed, Amelα7 was also expressed in the antennal lobes and optic lobes. Expression in the MBs was found in the outer and non-compact Kenyon cells (Thany et al., 2005).

We then studied the expression of the Pameα7 subunit in the *Xenopus laevis* oocytes and found that it can form functional homomeric receptors in the *Xenopus laevis* oocytes. To date functional expressions of an insect homomeric α receptor had been demonstrated in *Xenopus laevis* oocytes with the *Drosophila melanogaster* Dα5 and Dα7 subunits co-expressed with the molecular chaperone CeRIC-3. Our investigations suggest that the cockroach Pameα7 receptor is insensitive to α-Bgt. This result was not surprising as there was also a lack of specific α-Bgt binding sites on drosophila S2 cells, expressing full-length Dα6 or Dα7 subunits. The only exception were the use of chimeric Dα6/5HT_3A_ and Dα7/5HT_3A_ receptors or recombinant Dα6 and Dα7 receptors expressed with RIC-3 (Lansdell and Millar, 2004; Lansdell et al., 2012). Moreover, cockroach *Periplaneta americana* did not express an α5 subunit compared to other insect species such as *Drosophila melanogaster*. Similar lack of α5 subunit ortholog was also shown in the pea aphid *Acyrthosiphon pisum* (Dale et al., 2010). The lack of this subunit may impact the expression and the functional properties of α7 subunit because it can form an heteromeric receptor with α7 subunit as found with *Drosophila melanogaster*.

In conclusion, in the present study, the challenge was to identify a cockroach Pameα7 subunit which was able to express functional receptor from direct expression in the *Xenopus* oocytes. But, we are aware that additional efforts are needed because we have to consider that currents are low when we use low acetylcholine and nicotine concentrations. Indeed, at low concentration, we did not find endogenous responses following application of nicotine (at 1 mM). At high nicotine (5 and 10 mM) concentrations, despite that currents are high, endogenous currents were found which lead us to be care on the results. Nevertheless, with all due caution, we consider that Pameα7 subunit can form functional homomeric receptor. Moreover, The low sensitivity could suggest that Pameα7 needs cockroach chaperone proteins like RIC-3 or NACHO (Gu et al., 2016). We have started to clone cockroach orthologs of RIC-3 and the nAChR regulator, NACHO which we hope will help increasing currents through cockroach Pameα7 receptors. Indeed, despite that the mammalian RIC-3 increases α7 activity, it is not sufficient for efficient assembly of α7 but NACHO can synergize with RIC-3 for α7-type nAChRs surface expression (Matta et al., 2017). In addition to the cloning of RIC-3 and NACHO, we aim to study the involvement of lynx proteins identified in *Locusta migratoria*, in particular lynx3 which increased epibatidine-evoked current amplitudes when it was co-expressed with both Locα1 and rat β2 or Locα4 and rat β2 in the *Xenopus* oocytes (Bao et al., 2017). All these studies will be our future goal.

## Data Availability Statement

The datasets generated for this study can be found in the GenBank accession number: JX466891.

## Ethics Statement

All the experiments were performed with laboratory-reared insect. No special permit was required. All European guidelines for the care and use of laboratory animals were followed.

## Author Contributions

ST and J-YL designed the experiments. AC, ET, and CM performed the experiments. AC, ET, BS, JG, J-YL, and ST analyzed the data and wrote the manuscript.

## Conflict of Interest

The authors declare that the research was conducted in the absence of any commercial or financial relationships that could be construed as a potential conflict of interest.

## Footnotes

1 http://www.ncbi.nlm.nih.gov/genbank/
