# Corrigendum: Cloning and expression of cockroach α7 nicotinic acetylcholine receptor subunit

**DOI:** 10.3389/fphys.2024.1168531

**Published:** 2024-05-30

**Authors:** Alison Cartereau, Emiliane Taillebois, Balaji Selvam, Carine Martin, Jérôme Graton, Jean-Yves Le Questel, Steeve H. Thany

**Affiliations:** ^1^ LBLGC, UPRES EA 1207-USC INRA 1328, Université d’Orléans, Orléans, France; ^2^ Roger Adams Laboratory, University of Illinois at Urbana-Champaign, Urbana, IL, United States; ^3^ CEISAM-UMR CNRS 6230, Faculté des Sciences et des Techniques, Université de Nantes, Nantes, France

**Keywords:** nicotinic receptor, insect, α7 subunit, acetylcholine, nicotine, neonicotinoid

In the published article, there were some errors in **Supplementary Figures A, B** and their legend. Figure A was the wrong figure, while Figure B contained a mistake. It indicated 10 cycles in the first histograms, while the correct number is 15. The legend indicated that no significant amplification was found at 10 and 15 cycles except for actin (figure A). The correct statement is “No significant amplification was found at 10 cycles except for actin (figure A)”. The correct figures A and B and their legend appear in the updated Supplementary Material.

The authors apologize for these errors and state that this does not change the scientific conclusions of the article in any way. The original article has been updated.

