# Supplementary figures and images for: Cloning and Expression of Cockroach α7 Nicotinic Acetylcholine Receptor Subunit
Source: Front Physiol. 2020 May 7;11:418. doi: 10.3389/fphys.2020.00418 (PMC7221154; doi:10.3389/fphys.2020.00418)

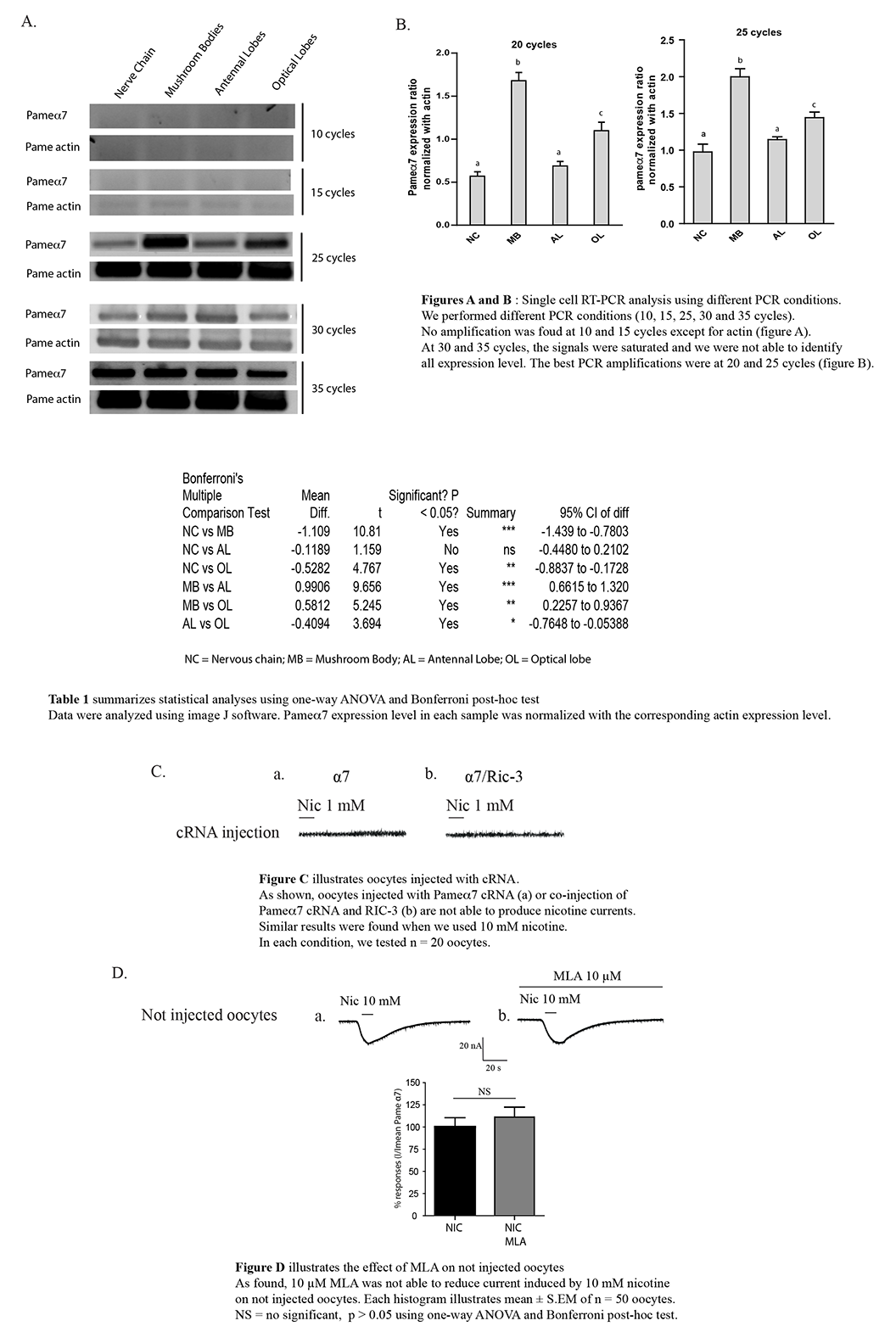

Supplement: Supplementary file 1 [file Image_1.tif]
